# Supplementary material for: Platelet-Rich Plasma (PRP) Based on Simple and Efficient Integrated Preparation Precises Quantitatively for Skin Wound Repair
Source: Int J Mol Sci. 2024 Aug 28;25(17):9340. doi: 10.3390/ijms25179340 (PMC11394848; doi:10.3390/ijms25179340)
Supplement: Supplementary file 1 [file ijms-25-09340-s001.zip › ijms-3113578-supplementary.pdf]

Supporting Information

Table S1. Box–Behnken design for the independent variables and corresponding response values

No. of runs	X_1: RCF (×g)	X_2: Time (min)	X_3: Separation gel content (g)	Platelet recovery rate (%)
1	1500	10	1	83.17±1.23
2	2000	5	1	75.23±1.47
3	1500	15	0.5	76.94±0.51
4	1000	15	1	55.10±0.49
5	1000	10	1.5	64.41±1.20
6	1500	10	1	84.97±0.82
7	1500	10	1	86.35±1.02
8	1500	5	1.5	94.12±0.75
9	1500	10	1	80.94±0.58
10	1500	10	1	87.11±0.08
11	2000	10	0.5	56.26±0.34
12	1500	5	0.5	63.15±0.44
13	1500	15	1.5	84.97±0.15
14	2000	10	1.5	90.19±0.11
15	1000	5	1	60.22±0.07
16	1000	10	0.5	49.04±0.06
17	2000	15	1	84.07±0.10

Table S2 Fit statistics

**Std. Dev.**	2.76	**R^2**	0.9831
**Mean**	75.07	**Adjusted R^2**	0.9613
**C.V. %**	3.68	**Predicted R^2**	0.8432
		**Adeq Precision**	21.9964

Table S3 Regression coefficient (β), coefficient of determination and F test value of the predicted second order polynomial models for platelet recovery rate

**Factor**	**Coefficient (β)**
Intercept	84.40
Linear	
X_1	9.63***
X_2	1.25
X_3	-11.13***
Quadratic	
X_1^2	-15.58***
X_2^2	-0.32
X_3^2	-4.07*
Cross product	
X_1X_2	3.50*
X_1X_3	-4.75***
X_2X_3	5.50**
R^2	0.9831
Adj. R^2	0.9613
F value (model)	47.51***
F value (lack of fit)	1.48

X_1 Relative centrifugal force (×g), X_2 centrifugation time (min), X_3 serum separation gel content (g), R^2 coefficient ofdetermination Level of significance: *$p < 0.05$, **$p < 0.01$, ***$p < 0.001$

Table S4 Model test - platelet recovery rate

Groups	PLT recovery rate (%)
1	96.14
2	94.02
3	96.10
4	96.76
5	95.70
Mean	95.74
RSD	1.04

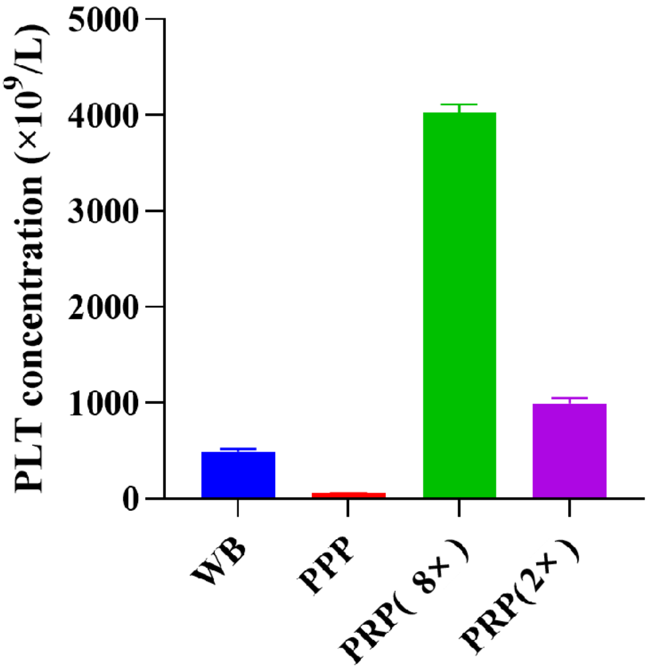

Figure S1. PLT concentration of PRP and PPP.
